# An Intelligent Gesture Classification Model for Domestic Wheelchair Navigation with Gesture Variance Compensation

**DOI:** 10.1155/2020/9160528

**Published:** 2020-01-30

**Authors:** H. M. Ravindu T. Bandara, K. S. Priyanayana, A. G. Buddhika P. Jayasekara, D. P. Chandima, R. A. R. C. Gopura

**Affiliations:** ^1^Intelligent Service Robotic Group, Department of Electrical Engineering, University of Moratuwa, Moratuwa 10400, Sri Lanka; ^2^Bionics Laboratory, Department of Mechanical Engineering, University of Moratuwa, Moratuwa 10400, Sri Lanka

## Abstract

Elderly and disabled population is rapidly increasing. It is important to uplift their living standards by improving the confidence towards daily activities. Navigation is an important task, most elderly and disabled people need assistance with. Replacing human assistance with an intelligent system which is capable of assisting human navigation via wheelchair systems is an effective solution. Hand gestures are often used in navigation systems. However, those systems do not possess the capability to accurately identify gesture variances. Therefore, this paper proposes a method to create an intelligent gesture classification system with a gesture model which was built based on human studies for every essential motion in domestic navigation with hand gesture variance compensation capability. Experiments have been carried out to evaluate user remembering and recalling capability and adaptability towards the gesture model. Dynamic Gesture Identification Module (DGIM), Static Gesture Identification Module (SGIM), and Gesture Clarifier (GC) have been introduced in order to identify gesture commands. The proposed system was analyzed for system accuracy and precision using results of the experiments conducted with human users. Accuracy of the intelligent system was determined with the use of confusion matrix. Further, those results were analyzed using Cohen's kappa analysis in which overall accuracy, misclassification rate, precision, and Cohen's kappa values were calculated.

## 1. Introduction

Assistive technology for elderly and disabled people is an expeditiously growing field [1, 2]. Many researches are focused on edifying living standards of human life. Common issue with most elderly and disabled persons is navigation. Since it is hard for them to move around, they need some assistance from another person or a machine. However, assisting is not sufficient when there are communication problems [3]. It is hard to navigate in a domestic environment with difficulties to communicate accurately. Disabled and elderly people are increasingly observed with speech disorders such as apraxia of speech, stuttering, and dysarthrias. Hence, vocal interaction becomes difficult to those among this community. Moreover, incapability to navigate in a domestic environment without getting help creates common issues like anxiety, anger, and depression which leads to poor health conditions [4]. Therefore, it is obvious that assistive technologies should upgrade in a more intelligent manner to make human life more comfortable and healthier [5].

Human prefers to use multiple modalities such as voice and gestures to interact with each other in a domestic environment [6–8]. Gestures included hand gestures, facial expressions, or cues which are difficult to understand even for human beings. Furthermore, vocal and gestural expressions can be integrated to create navigation commands [9]. As an example, a person in a wheelchair might say “go there” and the person can integrate hand gesture to the sentence by showing which direction that he wants to move [10]. Vocal command may contain uncertain terms such as distance and direction expressing terms like “near,” “far,” “middle,” “left,” and “right” or place expressing terms like “here” and “there” [11]. Interpreting uncertain terms are a difficult task for a robot. Moreover, such interpretation depends on various factors such as user experience, eyesight, environment, and cognitive feedbacks from the environment [12–14]. Consideration of those factors makes vocal command interpretation extremely difficult. However, using an intelligent system with a capability to understand such phrases can be unlikely to be used by humans because small error or misinterpretation made by the system can do critical damage to a disabled or elderly human being.

Most elderly people show speech difficulties which make it difficult to clarify voice commands given by them using speech recognition systems. Moreover, voice commands include various types of uncertainties such as time related, frequency, distance, and direction which make it hard to understand. As an example, “go there” and “come here” have position uncertainty and commands like “wait here” and “give me a minute” have time-related uncertainty. Gestures are widely used in navigation and also have variances [15]. However, comparing to vocal commands, gestures show a more detailed instruction which lead to a more accurate decision of an intelligent system. If an intelligent system is capable of interpreting gestures into navigation commands and a person could give navigation commands using hand gestures for all the essential navigation tasks, it will be a more simple and efficient method for systems like intelligent wheelchairs [16]. Such systems should possess a capability to interpret hand gestures while understanding variances and would be like gesture alphabet for navigation. Moreover, those gestures should be easily remembered and able to express every essential task that may be required by a disabled or elderly person on a wheelchair. Furthermore, misunderstanding of unintended hand movements can create critical situations. Therefore, safety precautions should be also considered [17].

A method has been proposed in [18] to recognize dynamic hand gestures of human hand using a RGB depth camera. The system is capable of automatically recognizing hand gestures against a complicated background. However, the system is capable of identifying limited navigation commands, and the system is designed to control a mobile robot using hand gestures. Therefore, the robot can perform only the basic robot motions. A real-time hands-free immersive image navigation system that can respond to various gestures and voice commands has been proposed in [19]. The system has a capability to identify a wide range of hand and finger gestures and voice commands using Kinect and leap motion sensors. However, the system is specifically designed for image navigation, and it does not possess any motion navigation understanding capability.

An intelligent wheelchair with hand gesture recognition facility is developed in [20]. The wheelchair can be controlled through basic hand gestures such as FORWARD, BACKWARD, and RIGHT/LEFT. However, this wheelchair is not capable of recognizing more complex static and dynamic gestures, and recognition of tasks is not in real time.

Another method has been proposed in [21] to recognize dynamic hand gestures using a leap motion sensor. This system can recognize simple dynamic gestures such as swipe, tap, and drawing circle to authenticate logins. However, this system cannot recognize complex dynamic gestures used in a domestic navigation task. A dynamic and static gesture recognition method has been proposed in [22] for an assistive robot. This system can recognize simple dynamic gestures such as waving and nodding while simple pointed gestures can be identified in locating places. However, this system cannot recognize dynamic motion commands of hand and static commands used for navigation. Another weakness of this system is the lack of flexibility in using separate fingers and the lack of real-time gesture recognition. Most dynamic and static gestures use separate fingers.

The hand recognition system proposed in [23] is using a Kinect sensor to get the depth map and the color map. The use of the depth map with a color map has increased the robustness of the gesture recognition, and the Finger-Earth Mover's Distance method has been used to remove any input variation or the color distortions. As this method only considers distance between fingers, movement of fingers against each other will not be detected. These types of finger tremors cause gesture variances which will not be recognized in this setup. The purpose of this article is to develop a simple yet unique gesture system to help navigate in domestic environments compensating above-mentioned gesture variances. The method proposed in [24] has used depth image to identify real-time dynamic hand gestures through a Hidden Markov Model (HMM). Dynamic hand gesture variances considering hand orientations, speed, and styles have been considered in this system. However, miniscule variances such as finger orientations, finger bone orientations, and finger speeds have not been considered in this system. There is another method that has used the HMM to space-time hand movement pattern in a 3D space [25]. In this method, they have considered hand movement, palm orientation in a 3D space to compensate for the hand gesture variances or tremors. However, it fails to identify the finger movements against the palm orientation usually seen among elderly. There are many hand gesture recognition systems that have been developed in order to recognize most static and dynamic hand gestures. However, very few have tried to compensate the involuntary hand gesture variance. Systems introduced in [26, 27] have tried to define more features in order to minimize all static and dynamic variances or tremors. To avoid overfitting and redundancy, they have used 2 level classifier fusion to filter out the unnecessary features. Even with about 44 features, individual classifiers, and 2 level fusions, the system in [26] has failed to compensate the finger tremors. Since they [26, 27] have not considered finger angles against the palm orientation or bone angles, fusion of those features into their methods become tediously difficult. The system developed in [28] has introduced a gesture vocabulary to operate a mobile phone as opposed to the system proposed in this article. However, this system has considered both large scale hand gestures and small scale gestures in which miniscule gesture variances matter. Bayesian linear classifier has been used in small scale gestures while HMMs have been used in large scale gestures. However, finger movements, bone angles, or finger orientations which were not considered in the features and variances in both static and dynamic gestures will not be compensated by using individual classifiers.

Therefore, this paper presents a novel method to recognize dynamic and static motion-related hand gestures even with tremors, based on a gesture classification model for wheelchair users with speech disorders. A complete gesture model with essential navigation commands is defined. It can be used to navigate an intelligent wheelchair through a domestic environment. Elaborated feature set is extracted in order to compensate for user variances that occur in gestures.

The purpose of this article is to develop a hand gesture model to help a wheelchair user to navigate in a domestic environment. Therefore, the gestures designed have to cover all possible navigation scenarios. These gestures will vary as static, dynamic, palm, and finger gestures. A system should be able to recognize not only both static and dynamic gestures, but it should be able to compensate hand and finger tremors happening among elderly. A system should be able to identify different variations of the same gesture from one user to the other. In summary, none of the above existing systems was not specifically designed as a gesture model for navigation. There were few which worked as a sign language gesture model. However, those gesture vocabularies will not be effective for the purpose of this article. Gesture recognition methods and tools used in the above systems have focused in the accuracy of a gesture. Some systems have considered gesture variances caused by palm tremors. However, none of them has considered finger tremors and finger bone angles as possibilities. In this article, we are not only focusing on developing a specifically designed gesture vocabulary but also considering all possible variations of the same gesture.

Therefore, tremors in the elderly people will not be a cause of confusion for the navigation system. The overall functionality of the proposed system is explained in Section 2. The proposed concept to create a gesture model and feature extraction process is explained in Section 3. Experimental results are presented and discussed in Section 4. Finally, the conclusion is presented in Section 5.

## 2. System Overview

**Figure 1:**
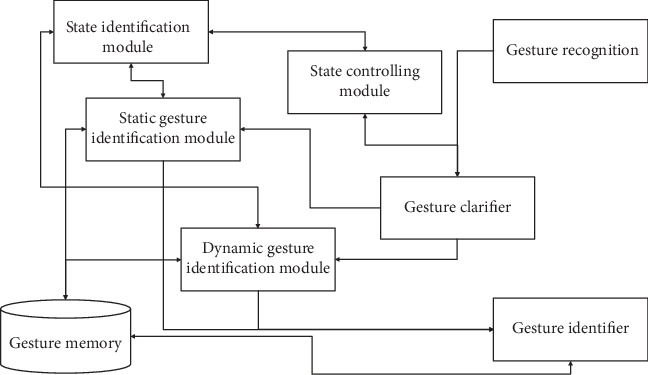
System overview.

Overall functionality of the proposed system is shown in Figure 1. The proposed system is capable of identifying static and dynamic gestures and interpreting those gestures into navigation commands. Gesture Memory (GM) is built based on identified gestures from a human study which are capable of creating every essential navigation task in a domestic environment. Moreover, user's capability to remember and recall gestures is also evaluated.

Gesture recognition module extracts the information of hand skeleton using a leap motion sensor, and extracted data is sent to the Gesture Clarifier (GC) for clarification of gestures into static and dynamic gestures based on gesture features. Static Gesture Identification Module (SGIM) and Dynamic Gesture Identification Module (DGIM) understand and identify the navigation command related to the observed gesture. State Identification Module (SIM) works together with GC, SGIM, DGIM, and State Controlling Module (SCM) in order to differentiate gestures and unintended hand movements. SCM understands user requirement to use a gesture identification system by controlling most prioritized gesture commands such as “Turn on” and “Turn off.”

## 3. Gesture Model

### 3.1. Human Study I: Identification of Navigation Commands

**Figure 2:**
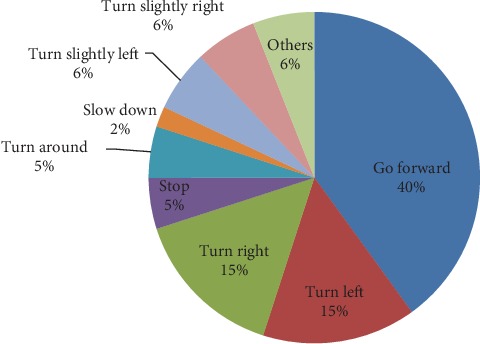
Frequency of navigation commands.

Natural human communication consists of multiple modalities like voice and hand gestures. Therefore, defined hand gestures should have been able to replace all possible navigational commands. In order to identify the commands used by wheelchair users during basic navigation, a human study was conducted. 20 wheelchair users of age 55 to 70 have participated in the study. Participants were asked to guide their wheelchair using hand gestures or voice or multimodal interaction. Natural navigation command identification was the priority. Hence, interaction method was not limited to hand gestures. Location is changed in order to cover all possible navigation scenarios. Participants did not have any prior knowledge of the locations or the previous study results. Hence, the accuracy of the results was ensured, and repetition of results was avoided. All possible navigation commands were recorded, most frequent commands were identified, and the graphical representation of the identified command frequencies is given in Figure 2.

Most frequent commands identified above were considered for the proposed gesture system.

### 3.2. Human Study II: Hand Gesture Identification

A human study was conducted in order to understand the hand gesture features used by wheelchair users for the identified navigational commands. A group of 20 people randomly selected from the same age group (55 to 70) participated in the study. Participants were asked to execute the basic navigation commands, identified in the human study I using only the hand gestures. Data collected in this study were used to build the gesture system that will be elaborated later. A leap motion sensor was used to track hand gestures, and raw data collected through that were processed to identify the gesture features. Most predominant hand feature associated in executing each command was recorded. Results are shown in Table 1. Frequently used hand features for each gesture were used as a basis in feature extraction.

Two main types of hand gestures were identified as static gestures (pointers or poses) and dynamic gestures (hand movements). Static gestures were mainly used in subtle motion commands like Stop, Turn around, and Turn slightly left/right. For vigorous motion commands, participants used hand movements. Other important tendency was that participants liked to use both static and dynamic gestures more evenly. These commands are also found to be two types: finger-pointing gestures and palm-opening gestures. Numbers of fingers used by the participants were unpredictable in pointing gestures, and mainly one finger or two fingers were used. Dynamic gestures were mainly used to express movements and directions that a wheelchair needs to execute.

### 3.3. Hand Gesture System

**Figure 3:**
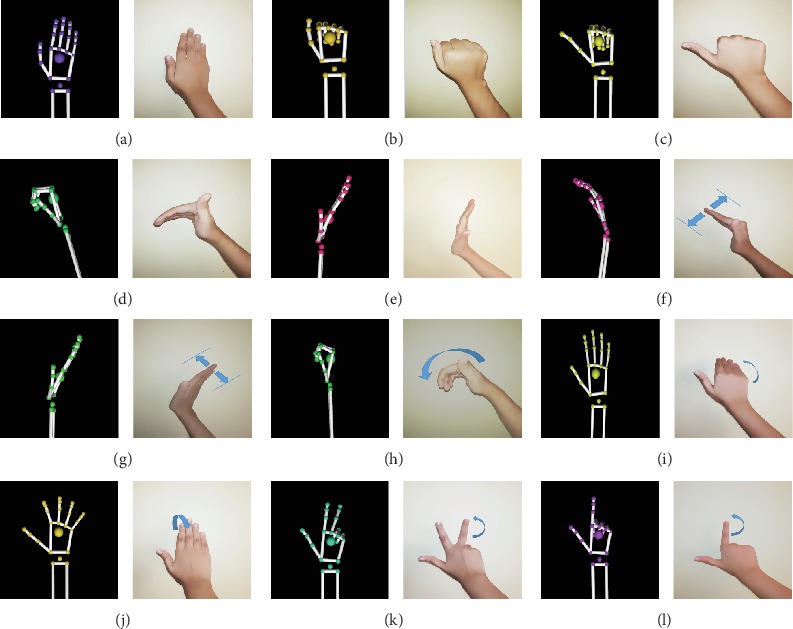
Navigation gestures: (a) Go forward, (b) Stop, (c) Go backward, (d) Hard left, (e) Hard right, (f) slightly left, (g) Slightly right, (h) Turn around, (i) Slow down, (j) Go faster, (k) Turn off, and (l) Turn on.

Navigation commands of a wheelchair user should cover all the possible navigation scenarios. If the user has vocal abilities, the commands will include information covering exact instructions. For an intelligent wheelchair to work through only hand gestures, they should be simple, clear, and accurate. The proposed gesture system is based on all basic navigation scenarios. These hand gestures are simple and clear. Out of the hand gestures defined, dynamic gestures were used to represent motion instructions. Defined hand gestures are given in Figure 3.

The gesture system was built based on the following considerations.

1. Defined hand gestures should be simple, clear, and accurate
2. Gestures should be defined in a way that a user can navigate through a path using a minimum number of gestures
3. A user should be able to remember and recall the defined hand gestures. To ensure user's adjustability to the gesture system, a human study was conducted. Details of this study are explained in Sections 4.1 and 4.2
4. Significant difference should be identified among hand gestures. Therefore, users will not have any confusion with gestures
5. A hand gesture system should have both static and dynamic gestures in order to mitigate inaccuracies caused by the leap motion sensor

### 3.4. Feature Extractions

Hand gestures accompanied with vocal interaction tend to be both voluntary and involuntary. These gestures carry information such as direction and motion. For a wheelchair user with vocal disabilities, these hand gestures could be considered as the primary modality. Even though there are gesture systems such as American sign language, the execution of these gestures differs from one elderly person to the other. To compensate for this variation, bone angles as explained below were used.

Defined gesture system consists of two main forms of gestures: dynamic gestures and static gestures. Static gestures are nonmoving hand poses which can be modeled through basic hand features. Dynamic gestures are modeled using dynamic hand features like finger movement and hand movement.

**Figure 4:**
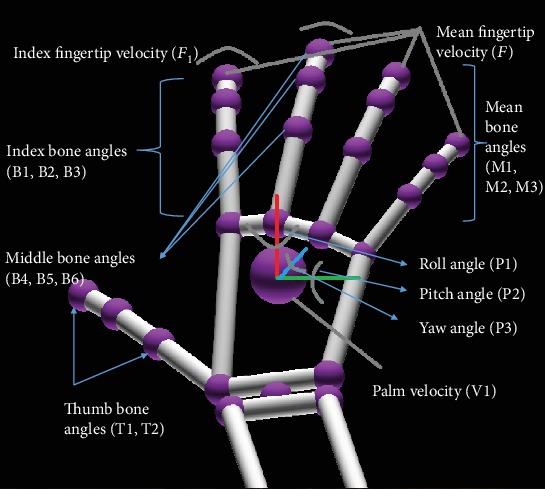
Features extracted from hand skeleton.

**Figure 5:**
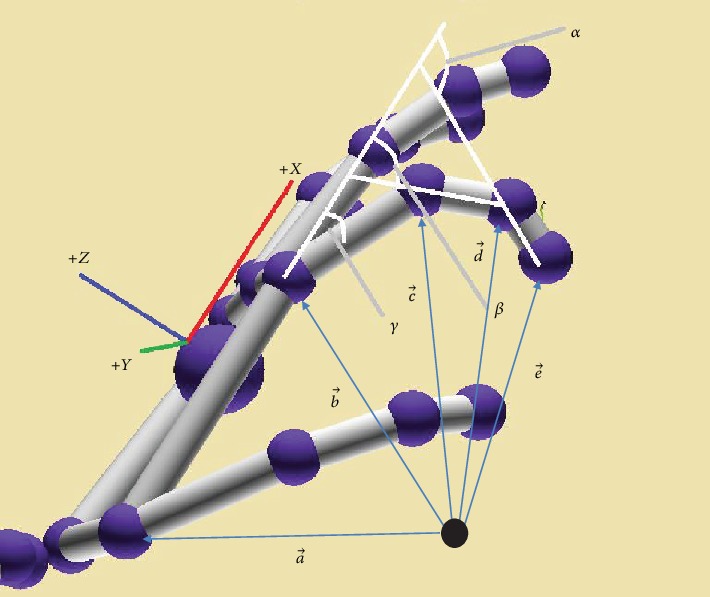
Hand features.

1. *Palm orientation.* Palm orientation was taken based on leap motion coordinates. The pitch angle, roll angle, and yaw angle of the palm depict the orientation. Pitch angle is the angle rotated around the +*Y* axis, roll angle is the angle rotated around the +*X* axis, and Yaw angle is the angle rotated around the +*Z* axis. As illustrated in Figure 4, the Quaternion angle theory is used to take the yaw, pitch, and roll of the *x*, *y*, *z* vectors relative to a single vector. Usually Euler angles are used as it has the ability to take the vectors relative to each other. But Euler angles have certain limitation that can be addressed by Quaternion angles. The main limitation of using Euler angles is that difficulty in interpolating between two orientations of an object smoothly [29]
2. *Finger bone angles.* Bone angles of fingers with respect to the metacarpal bone of the hand are extracted. These angles are shown in Figure 5. Hence, even when (i) Slow down, (j) Go faster, (k) Turn off, and (l) Turn on, fingers have improper position that will not affect the gesture recognition. As shown in Figure 5, the angles of distal (*α*), proximal (*β*), and intermediate (*γ*) bones with respect to the metacarpal bone were calculated using Equation (1). These angles were taken for the index finger and middle finger. For the thumb finger, only the distal and proximal bone angles were taken as the thumb does not have an intermediate bone. These three fingers were considered specifically since most of the navigational gestures identified were associated with them. As the ring finger and pinky finger are tightly associated couple, the average of distal, proximal, and intermediate angles was considered. Navigational gestures defined have sole ring or pinky finger features. But it was important to get separate features for other three fingers as they were included separately in the hand gestures. Here, the direction of metacarpal bone, proximal bone, intermediate bone, and distal bone is denoted by $\vec{p}$, $\vec{q}$, $\vec{r}$, and $\vec{u}$, respectively.

(1) $$\begin{array}{c} \begin{array}{c} \vec{p}=\vec{b}-\vec{a},\\ \vec{q}=\vec{c}-\vec{b},\\ \vec{r}=\vec{d}-\vec{c},\\ \vec{u}=\vec{e}-\vec{d},\\ \alpha =\cos^{-1}\frac{\vec{u}\cdot \vec{p}}{\left|\vec{u}\left\|\cdot \right\|\left.\vec{p}\right\|\right.}. \end{array} \end{array}$$

The calculation of other two angles was done using the same approach

(3) *Fingertip velocity*. To detect the dynamic gestures defined, fingertip velocity of the index finger was considered. Two different inputs were considered for both magnitude and direction of the velocity vectors. Also, mean fingertip velocity of other fingers was considered to detect finger movements. All the properties considered are shown in Figure 4
(4) *Palm velocity*. To detect the palm movement of the hand, palm velocity magnitude and direction were considered as inputs. Palm orientation angles were also input features to detect dynamic gestures

### 3.5. Gesture Classification

Artificial Neural Networks (ANNs) have been developed to identify and clarify dynamic and static gestures. Each Static Gesture Identification Module (SGIM) and Dynamic Gesture Identification Module (DGIM) consist of an ANN. Gesture Clarifier (GC) consists of Algorithms 1 and 2 to distinguish dynamic gestures from static gestures. GC priorities dynamic gestures since critical commands like “Turn off” and “Turn on” are defined in DGIM. It controls system state based on prioritized commands. If received navigation command was “Turn off,” the GC will isolate GI from DGIM and SGIM and wait for the next command to be “Turn on.” Moreover, when a gesture confirmation is identified by GC and SCM, the appropriate submodule will be activated.

SGIM consists of an ANN that has 14 inputs (B1, B2, B3, B4, B5, B6, M1, M2, M3, T1, T2, P1, P2, and P3). There are two hidden layers in that ANN, and the output layer has four outputs (N1, N2, N3, and N4). The output of the SGIM represents a static navigation command number from 1 to 12. DGIM consists of an ANN that has 5 inputs (C1, C2, Q1, Q2, P1, P2, P3, and V1) and 4 outputs (N5, N6, N7, and N8). The output of the DGIM represents a dynamic navigation command number from 1 to 12. Both outputs of the SGIM and DGIM were in binary numbers. Both ANNs use a sigmoidal function as the activation function.

## 4. Result and Discussion

**Figure 6:**
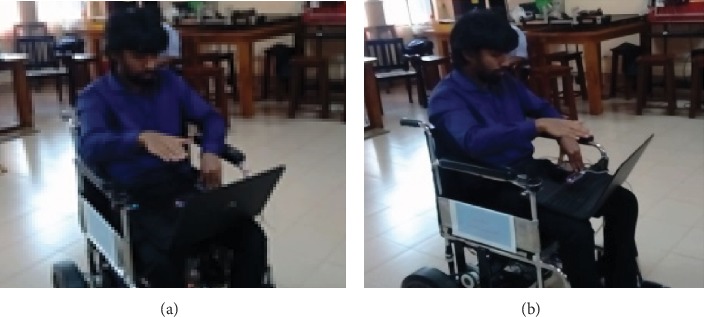
Research platform during experiment session.

To evaluate the validity and accuracy of the proposed intelligent gesture system, experiments were carried out from two aspects: (a) accuracy of remembering and recalling of the defined gestures and (b) accuracy and robustness of the intelligent hand gesture recognition system. System was implemented on the intelligent wheelchair explained in [20]. To carry out the experiments, a group of participants of 20 wheelchair users were randomly selected. They were selected from three age groups of 20 to 30, 30 to 55, and over 55 years. Participants were generally healthy with no cognitive impairments except for mobility impairment of legs. The research platform during the experiment session is shown in Figures 6(a) and 6(b).

The implementation of the developed intelligent system requires a high-performing smart wheelchair with fast and reliable computing power. For this purpose, we used a wheelchair robot which is developed in our laboratory that has basic navigational capabilities. In this wheelchair, we have installed an industrial grade high-end computer in which DDR4 SO-DIMM memory is 32 GB and processor is a 6th generation i7 quad core (3.6 GHZ). Also, to increase the computational capacity, a SSD memory of 1 TB is installed. To compensate for the high performance and rugged operation, it can withstand from -20°C to 60°C temperature. These are essential for the intelligent system to work properly since training and execution will take a lot of computational power.

### 4.1. Experiment I

**Figure 7:**
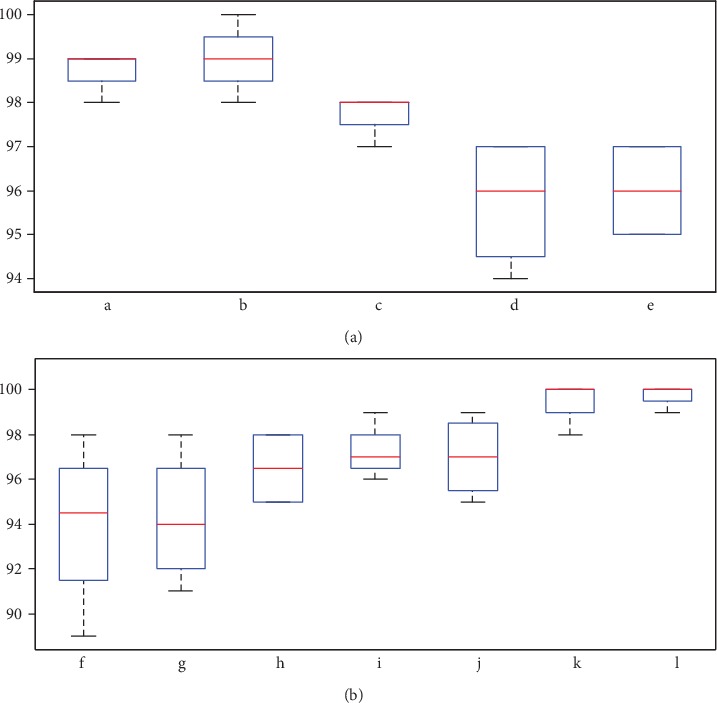
Boxplots to represent remembering and recalling capability of each dynamic and static hand gesture.

A detailed presentation of the navigation commands and relevant hand gestures was shown to each participant. They were asked to memorize the commands and gestures for 15 minutes. Then, each participant was asked to recall the relevant hand gesture for randomly given navigation command. Percentage accuracy of recalling the hand gesture for each navigation command was recorded as Exp. 1. In the next step, each participant was asked to recall the navigation command for a randomly given hand gesture. Percentage accuracy of recalling the navigation command for each hand gesture was recorded as Exp. 2. After that, participants were asked to recall all the navigation commands in one go. Percentage accuracy of recalling a navigation command was recorded for this step as Exp. 3. Finally, each participant was given a fixed navigation path, and they were asked to guide themselves with hand gestures defined in the system. Navigation path was selected considering all the navigation commands identified. Percentage accuracy of remembering each gesture in a task situation was recorded as Exp. 4. Recorded data is presented in Table 2. Boxplots given in Figures 7(a) and 7(b) show the remembering and recalling capability of each dynamic and static hand gesture.

### 4.2. Experiment II

**Figure 8:**
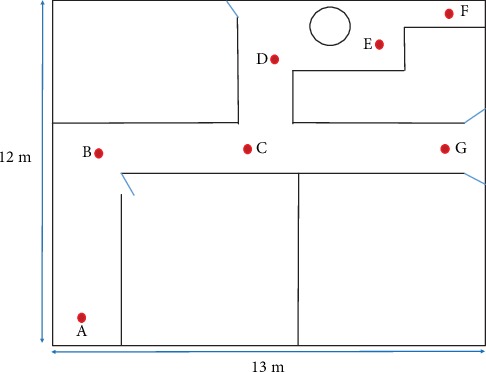
Experiment II setup. Participants were asked to give gesture instructions for the path A > B > C > D > E > F > E > D > C > G.

Participants were given a specific navigation task to complete using hand gestures. Navigation path of the task was planned in a way that all gestures were utilized. Navigation task and fixed path are given in Figure 8. Each participant had to guide themselves using the hand gestures, and the proposed system classified the hand gestures. This process was repeated for all the participants. System recognition accuracies were recorded for each hand gesture. Rates of success and failure in recognizing a particular hand gesture are given in the confusion matrix given in Tables 3–5.

In the experiment I, participants showed almost perfect memory of basic navigation commands such as “Go forward,” “Stop,” “Go backward,” and “Turn on/off” commands. Recalling accuracy percentage of most navigation commands was in the high 90s except for “Slightly right/left” commands. As mentioned in Table 2, Exp. 2 accuracies are higher than Exp. 1. Therefore, it can be deduced that recalling navigation command for hand gesture is easier. Exp. 4 values are slightly lower than other accuracy values. Recalling hand gestures during a task is tougher than in any situation. Overall, almost all accuracy values are higher than 90% and for most critical gestures such Turn on/off has almost perfect recalling accuracy. Therefore, it can be proved that the proposed gesture system is user friendly and easy to memorize.

In the experiment II, three confusion matrices were created in order to validate the recognition accuracies. For the two hand gesture recognition systems, static and dynamic, recognition accuracies were shown for each hand gesture.

For all the static gestures, accuracy is over 90% as shown in the confusion matrix given in Table 3. In the static gesture matrix, Cohen's kappa value was calculated with linear weighting. Used weights were equal in the static confusion matrix. For all the dynamic gestures, recognition accuracy is over 90% and overall accuracy is higher than the static gesture recognition system. Hence, the use of a high number of dynamic gestures than the static gestures for the system is validated. Cohen's kappa value was also calculated for this matrix as shown in Table 4 with linear weighting. Critical dynamic gestures such as “Turn on/off” were weighted with two points and other gestures with one point. Since kappa values for both recognition systems are over 0.81, it can be proved that the systems are working properly. For the gesture type selection system, a confusion matrix was created and overall accuracy, misclassification rate, precision, and Cohen's kappa values were calculated. Overall, accuracy is 0.94 (>0.90) and kappa value is over 0.81. Therefore, it can be concluded that selection system is also working properly.

## 5. Conclusions

This paper proposed a novel method to identify hand gestures related to navigation based on a gesture recognition model with compensations for user variances. An intelligent gesture identification system was introduced in order to clarify gestures with high precision. Bone angles with respect to metacarpal bone were introduced as novel features in order to elevate identification of gesture variances. The system is capable of eliminating complications due to user inability in executing precise hand gestures. An intelligent clarification system has been implemented to separate static and dynamic hand gestures. Experimental results confirmed that the wheelchair users with speech disabilities can remember and recall the proposed hand gesture system. Therefore, the proposed gesture model can be considered as user friendly, and it is concluded that the proposed intelligent gesture recognition system can recognize user hand gestures with a high accuracy.

## Figures and Tables

**Algorithm 1:**
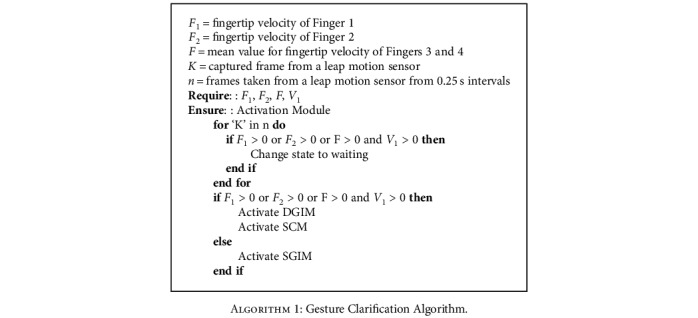
Gesture Clarification Algorithm.

**Algorithm 2:**
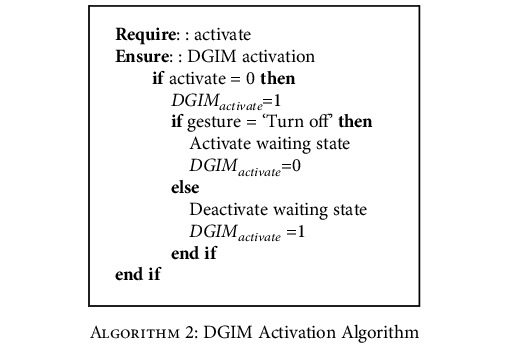
DGIM Activation Algorithm

**Table 1:** An analysis of hand feature frequencies associated with navigation commands.

Navigation command	Palm orientation	Palm movement	Fingertip movement	Finger bones	Fingers	
					Single finger	Multiple fingers
Go forward	92%	32%	28%	6%	44%	56%
Turn left	84%	75%	42%	18%	8%	48%
Turn right	82%	75%	44%	16%	7%	52%
Stop	96%	68%	64%	24%	18%	74%
Turn around	98%	56%	86%	77%	14%	84%
Slow down	98%	87%	54%	21%	19%	72%
Turn slightly left	90%	34%	97%	42%	47%	52%
Turn slightly right	89%	35%	98%	44%	46%	53%

**Table 2:** Result of experiment I.

Command no.	Navigation command	Exp. 1	Exp. 2	Exp. 3	Exp. 4
a	Go forward	98%	99%	99%	99%
b	Stop	99%	99%	98%	100%
c	Go backward	98%	98%	97%	98%
d	Hard left	95%	97%	97%	94%
e	Hard right	95%	97%	97%	95%
f	Slightly left	94%	95%	98%	89%
g	Slightly right	93%	95%	98%	91%
h	Turn around	95%	95%	98%	98%
i	Slow down	96%	97%	97%	99%
j	Go faster	95%	96%	98%	99%
k	Turn off	100%	100%	98%	100%
l	Turn on	99%	100%	100%	100%

**Table 3:** Confusion matrix for identification of static gestures.

	a	b	c	d	e
a	0.99			0.01	
b		0.97	0.03		
c		0.02	0.98		
d		0.01		0.99	
e	0.01				0.99
Observed kappa	Standard error	.95 confidence interval			
		Lower limit		Upper limit	
0.9648	0.0145	0.9363		0.9933	

**Table 4:** Confusion matrix for identification of dynamic gestures.

	f	g	h	i	j	k	l
f	0.95	0.01	0.04				
g	0.01	0.99					
h	0.04		0.96	0.01			
i			0.03	0.97			
j				0.03	0.97		
k						1.00	
l							1.00
Observed kappa		Standard error		.95 confidence interval			
				Lower limit		Upper limit	
0.9879		0.003		0.9819		0.9939	

**Table 5:** Confusion matrix for identification of dynamic and static gestures.

	Static	Dynamic	
Static	24	1	25
Dynamic	2	23	25
	26	24	
Accuracy			0.94
Misclassification rate			0.06
Precision			0.95
Cohen's kappa			0.88 (>0.81)

## Data Availability

The data used to support the findings of this study are included within the article.
